# Modelling Curved Contact Flexible Microstrip Applicators for Patient-Specific Superficial Hyperthermia Treatment Planning

**DOI:** 10.3390/cancers12030656

**Published:** 2020-03-11

**Authors:** H. Petra Kok, Jort Groen, Akke Bakker, Johannes Crezee

**Affiliations:** Department of Radiation Oncology, Amsterdam UMC, University of Amsterdam, 1105 AZ Amsterdam, The Netherlands; jort.groen@gmail.com (J.G.); akke.bakker@amsterdamumc.nl (A.B.); h.crezee@amsterdamumc.nl (J.C.)

**Keywords:** hyperthermia, electromagnetic, antenna, treatment planning, RF heating

## Abstract

This paper describes a method to reconstruct bendable superficial hyperthermia applicators for routine clinical patient-specific treatment planning. The reconstruction uses a CT scan with a flexible silicone dummy applicator positioned on the patient. The curvature was approximated by two second-degree polynomial functions. A realistic treatment series was mimicked using a standard Alderson radiation therapy phantom and a treatment planning model was reconstructed from a CT scan. The variation among treatment curvatures was compared to the modelled curvature. The mathematical approximation of the applicator curvature was validated for this phantom experiment, as well as for clinical treatments. The average maximum variation among the successive mimicked sessions was 3.67 ± 0.69 mm (range 2.98–4.60 mm). The maximum deviation between the treatment curvature and the modelled curvature was 4.35 mm. Comparing the treatment and approximated curvature yielded a maximum deviation between 2.98 mm and 4.12 mm. For clinical treatments the maximum deviation of the treatment and approximated curvature varied between 0.48 mm and 1.98 mm. These results allow adequate reconstruction of bendable hyperthermia applicators for treatment planning, which can further improve treatment quality, for example by optimizing the water bolus temperature for patient-specific tumor depths. Predictive parameters for hyperthermia treatment outcome can easily be evaluated and compared for various input parameters.

## 1. Introduction

Hyperthermia, i.e., heating of tumor tissue to 40–43 °C for 1 h, is a very effective treatment that enhances the effect of radiotherapy and chemotherapy [1]. Superficial hyperthermia combined with radiotherapy is a proven treatment combination for, e.g., recurrent breast cancer infiltrating up to 4 cm beyond the skin [2]. Randomized trials showed that the overall complete response rate for patients with recurrent breast cancer increases from 41% with radiation alone to 59% with radiation plus hyperthermia [2].

Accurate temperature information during superficial hyperthermia is essential to ensure treatment quality [3], and achieved temperatures are related to clinical outcome and thermal toxicity [4]. Information about the 3D temperature distribution is lacking, since standard temperature measurements during superficial hyperthermia are limited to measurements at the skin, combined with one or two invasive thermometry probes, if possible [3,5]. This makes it difficult to optimize temperatures during treatment and assess treatment quality, which supports the need for treatment planning in superficial hyperthermia.

Hyperthermia treatment planning simulates power absorption and/or temperature patterns in the patient to visualize the effect of different treatment strategies [6,7]. Treatment planning could be very helpful to obtain more information about the 3D temperature distribution and to optimize relevant treatment parameters, such as applicator positioning, bolus temperature and applied power. Different applicators can be compared to determine the most effective heating technique [8,9] and previous studies have demonstrated the usefulness of treatment planning for superficial hyperthermia [10], for example, to support the selection of the optimal superficial heating strategy in non-standard situations, such as when metallic implants are present in the heated region [11]. Scar tissue also requires special attention during treatment and in treatment planning, since hot spots (i.e., excessive normal tissue temperatures) can occur easily because of the low heat removal by perfusion at these locations [12].

**Figure 1:**
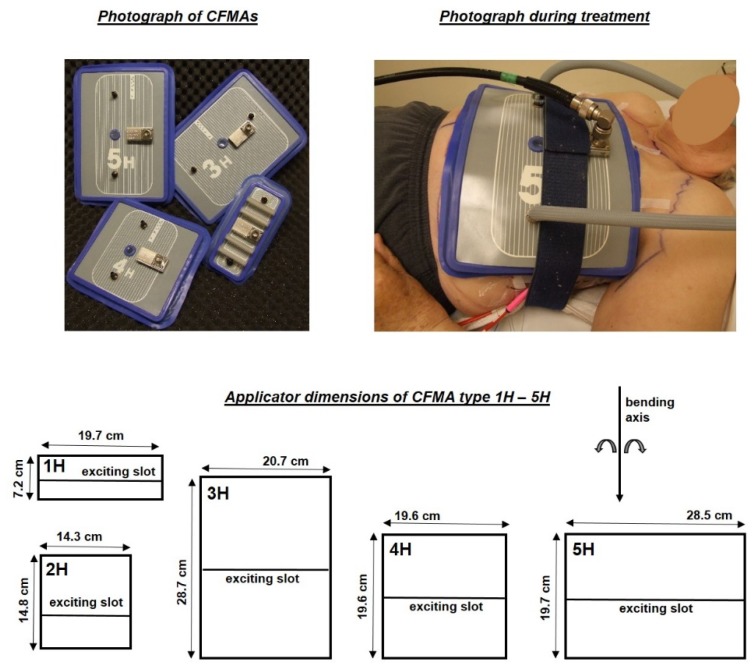
A photograph of four contact flexible microstrip applicators (CFMAs; top left), positioning during treatment (top right) and the five different sizes of CFMAs (bottom). The blue rubber frame around the antennas has an integrated water bolus for electromagnetic coupling and skin temperature control during treatment. The effective field size (i.e., the area covered by the 50% iso-SAR contour) is indicated by the white contour on the antennas.

Several different types of radiofrequency and microwave applicators are clinically used, with an operating frequency typically in the range of 434 MHz to 2450 MHz. Some of these designs are rigid, such as Lucite cone applicators, waveguides and spiral antennas [13,14,15,16]. Other applicators can either be bent or have a fixed curvature in order to follow the patient’s body contour, i.e., contact flexible microstrip applicators (CFMAs, Figure 1) and the ALBA4000 ON [17,18], respectively. Clinical advantages of these curved/bendable applicators are the body conformability and the increased effective heating depth (EHD), which depends on the curvature. The EHD is defined as the additional depth at which the relative power deposition has decreased to 50% of the value at 1 cm depth. The EHD is ~1.5 cm for a straight CFMA operating at 434 MHz [9,19]. With a mild curvature [20] (κ = 3.98 m^−1^), positioned at the top of an elliptical phantom (cross-section 36 × 25 cm), the EHD increases with 1–2 mm [19,21]. With a roughly three times sharper curvature (κ = 11.21 m^−1^), positioned around the side of the phantom, the increase is ~1 cm [19,21]. The shape of the applicator remains stable after bending.

Previous research validated modelling of standard straight and curved CFMAs by comparing measured and simulated specific absorption rate (SAR) distributions in tissue-equivalent phantoms [9,21]. When modelling elliptical phantoms, the curvature of the bent CFMAs can be described relatively easily using a partial ellipse. For patient-specific hyperthermia treatment planning a different approach is required to model the treatment position, orientation and the treatment curvature of the applicator. Linthorst et al. described a procedure to create a model for superficial hyperthermia with rigid Lucite cone applicators, using a mold representing the antenna footprint on the CT scan [22]. Drizdal et al. have developed a method to reconstruct the position of an array of six Lucite cone applicators from multiple-view images [23]. However, these methods were developed for straight applicators and would thus require adaptations to also reconstruct the antenna curvature, since modelling/rasterizing curved applicators for treatment planning requires a correct mathematical function describing the antenna curvature. Furthermore, since a number of manual steps are required, it takes about 1.5 h to reconstruct the applicator positions from a set of photos [23], which makes it less practical for routine clinical use. Methods to reconstruct curved superficial hyperthermia applicators for routine patient-specific treatment planning have not been described in the literature so far, and thus routine clinical application of treatment planning for superficial hyperthermia with curved applicators is not yet possible.

The aim of this study was to develop a new method to reconstruct the clinical applicator position, curvature and orientation of bendable hyperthermia applicators, which is suitable for routine clinical patient-specific hyperthermia treatment planning, i.e., applicator reconstruction should be possible within approximately 5 min. The developed method reconstructs the antenna position and shape based on specified points on a CT scan with a dummy applicator. Two separate second-degree polynomial functions are fitted to represent the applicator curvature. The curvature modelling is validated for CFMAs and the reconstruction method is applied on a CT scan of a patient with recurrent breast cancer to demonstrate the use in a clinical treatment planning process.

## 2. Materials and Methods

### 2.1. Contact Flexible Microstrip Applicators

**Figure 2:**
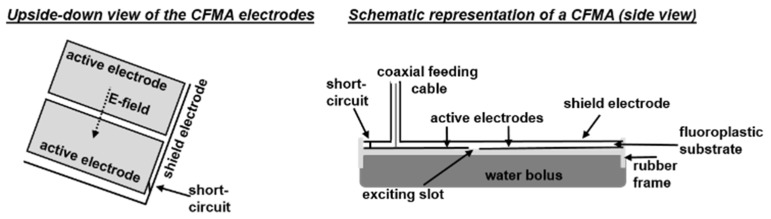
Schematic representation of a contact flexible microstrip applicator (CFMA).

CFMAs (SRPC “Istok”, Moscow, Russia) operating at 434 MHz are available in five different sizes and can be bent to follow the patient’s body contour [17]. For convenience a basic summary of the antenna design is given here. For more details the reader is referred to Gelvich and Mazokhin [17], who extensively described the design and technical aspects of the CFMAs. The five different sizes, labelled by the manufacturer as 1H (aperture size 7.2 × 19.7 cm), 2H (14.8 × 14.3 cm), 3H (28.7 × 20.7 cm), 4H (19.6 × 19.6 cm) and 5H (19.7 × 28.5 cm) are drawn schematically in Figure 1. The design of a CFMA is based on a quarter-wave microstrip resonator. A CFMA consists of two coplanar active electrodes and a shield electrode, with a fluoroplastic substrate in between [17]. The active electrodes are separated by means of an excitation slot of approximately 5 mm (manufacturer specifications). The orientation of the principal electric field component is perpendicular to this slot and bending is possible only around the axis perpendicular to the main field component. The microstrip line is excited by a center pin of a coaxial cable. A short-circuit is positioned at ~¼λ from the exciting slot (manufacturer specifications), where λ is the wavelength in the substrate [17]. A rubber frame surrounds the electrodes and this rubber frame has an integrated bolus bag filled with circulating deionized water to couple the electromagnetic fields into tissue and to control the skin temperature during treatment. Folds in the bolus should be prevented for optimal coupling and skin temperature control. A schematic drawing is shown in Figure 2.

### 2.2. Generation of a Patient and Applicator Model

**Figure 3:**
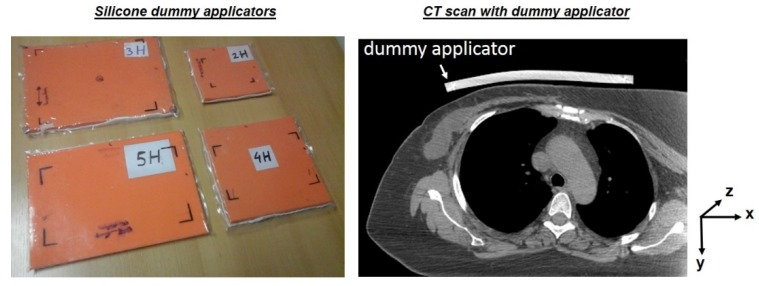
Photograph of four sizes of silicone dummy applicators (left) and a CT scan of a patient with a dummy applicator (right). As for the real antennas (Figure 1), the effective field size of the antenna is also marked on the dummy applicators.

A module-based software package for hyperthermia treatment planning (Plan2Heat) has been developed in C++ and validated at our department [24]. This work focuses on a method to model CFMAs in treatment position, which can be integrated into Plan2Heat. Hyperthermia treatment planning requires a patient model combined with a model of the hyperthermia applicator for simulation of the electromagnetic field, SAR and temperature distributions [6]. The patient model and the applicator model were reconstructed from a standard (hyperthermia) planning CT scan. Since the applicator contains metal structures causing scattering artefacts on the CT, flexible dummy applicators were created for each applicator size for use during the CT scan, to reconstruct the antenna orientation, position and curvature for treatment planning. The dummy applicators were made of silicone and the bi-component Eurosil 10 orange and Eurosil softener (Schouten group, Mijnsheerenland, The Netherlands) were used in mixing ratio 1:1:1. Depending on the tumor sizes, a dummy applicator was positioned on the patient according to the position, orientation and applicator curvature as during treatment (Figure 3), and attached with a Velcro strip as used during treatment (Figure 1). The CT scan was segmented into different tissue types based on Hounsfield Units [25], and dielectric and thermal tissue properties were assigned [26,27].

### 2.3. Applicator Curvature Estimation

**Figure 4:**
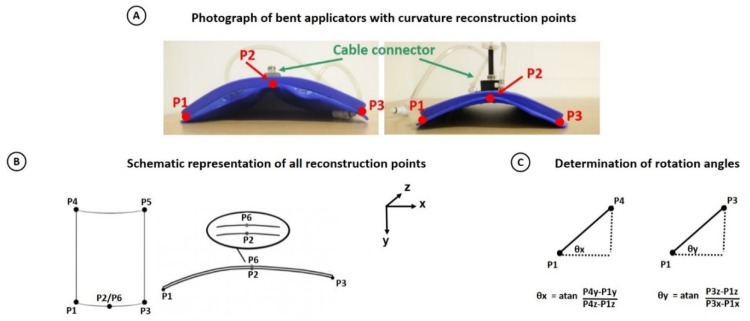
(**A**) Two different bent CFMAs with actual treatment curvature and curvature reconstruction points indicated. (**B**) Schematic representation of all reconstruction points P1–P6. (**C**) Determination of the rotation angles.

The first step of the planning process was to reconstruct the curvature of the applicator from the CT scan of the patient with the dummy applicator in the treatment position, to be described by an appropriate mathematical function. A translational symmetry along the z-axis was assumed, since the applicator is bent around the z-axis, which means that the curvature is assumed to be constant over the length of the applicator. Because of the rigid coaxial cable connector on top of the applicator the slope of the curve approaches zero around that location. This is illustrated by the two examples of bent applicators after a hyperthermia treatment shown Figure 4. Since the antenna follows the body contour, the curvature on the left and right side of the coaxial cable connector can be different. The function(s) describing the curvature of the antenna to be reconstructed should meet the following criteria:

- The slope is approximately zero near the rigid coaxial cable connector on top of the applicator.
- The reconstructed curve can consist of two segments, described by a different function.
- The areas with the strongest changes in slope are near the coaxial cable connector.

Given these criteria and the fact that the stiffness of the dummy and the real applicator may differ and thus the curvatures will not correspond exactly, using the exact contour of the dummy will not be feasible. To reconstruct a realistic applicator curvature from the CT scan, satisfying the above criteria, two functions are used to describe the curvature, one on each side from the connector. To this end, a starting point (P1), a center point (P2, where the slope approaches zero) and an end point (P3) were defined. A 2nd order polynomial function was fit through these user-defined points on the CT scan; two on the edges of the dummy and one where the slope of the curve approaches zero (Figure 4). This was used to describe the two parts, each defined by its own 2nd order polynomial function. Using the standard form of a polynomial function y = f(x) = Ax^2^ + Bx + C, we can define equations for the left hand side (subscripts L, Equations (1) and (2)) and the right hand side (subscripts R, Equations (3) and (4)):

(1) $$P1_{y}=A_{L}P1_{x}^{2}+B_{L}P1_{x}+C_{L},$$

(2) $$P2_{y}=A_{L}P2_{x}^{2}+B_{L}P2_{x}+C_{L},$$

(3) $$P2_{y}=A_{R}P2_{x}^{2}+B_{R}P2_{x}+C_{R},$$

(4) $$P3_{y}=A_{R}P3_{x}^{2}+B_{R}P3_{x}+C_{R},$$

Since the slope at the center of the curvature is zero, we have

(5) $$f^{\prime }\left(P2_{x}\right)=0,$$

which allows to derive *A_L_*, *A_R_*, *B_L_*, *B_R_*, *C_L_* and *C_R_*, defining the curve:

(6) $$A_{L}=\frac{P1_{y}-P2_{y}}{P2_{x}^{2}+P1_{x}^{2}-2\left(P2_{x}P1_{x}\right)},$$

(7) $$A_{R}=\frac{P2_{y}-P3_{y}}{P3_{x}^{2}+P2_{x}^{2}-2\left(P3_{x}P2_{x}\right)},$$

(8) $$B_{L}=-2A_{L}P2_{x},$$

(9) $$B_{R}=-2A_{R}P3_{x},$$

(10) $$C_{L}=P1_{y}-A_{L}P1_{x}^{2}-B_{L}P1_{x},$$

(11) $$C_{R}=P2_{y}-A_{R}P2_{x}^{2}-B_{R}P2_{x},$$

### 2.4. Curvature Recognition

Estimating both the x and y coordinate of P2 might introduce uncertainties in the reconstruction process, which can also result in incorrect reconstructed applicator dimensions. To ensure a robust reconstruction, only one coordinate of P2 was used in combination with the known applicator length using the equation

(12) $$\int_{P1_{x}}^{P2_{x}}dl=\int_{P1_{x}}^{P2_{x}}\sqrt{1+f^{\prime }{\left(x\right)}^{2}}\;dx\;=l_{P1P2},$$

where *l_P1P2_* is half the length of the applicator. A similar equation can be applied to the right half of the curve. Evaluating Equation (12) for *f(x) = Ax^2^ + Bx + C* yields

(13) $$\int dl=\;\frac{-\left(B-2Ax\right)\sqrt{{\left(B-2Ax\right)}^{2}+1}+arsinh\left(B-2Ax\right)}{4A}+constant,$$

where

(14) $$constant=\;\frac{-B\sqrt{B^{2}+1}+arsinh\left(B\right)}{4A},$$

since at x=0 the travelled distance is zero. Combining this with Equations (6), (8) and (10) yields a system of five non-linear equations for the left-hand side of the curvature profile:

(15) $$0=\;A_{L}P1_{x}^{2}+B_{L}P1_{x}+C_{L}-P1_{y},$$

(16) $$0=A_{L}P2_{x}^{2}+B_{L}P2_{x}+C_{L}-P2_{y},$$

(17) $$0=-2A_{L}P2_{x}-B_{L},$$

(18) $$0=\frac{-\sqrt{{\left(B_{L\;}-2A_{L}P2_{x}\right)}^{2}+1\;}\;\left(B_{L}-2A_{L}P2_{x}\right)+arsinh\left(B_{L}-2A_{L}P2_{x}\right)}{4A_{L}}+constant-l_{P1P2},$$

(19) $$0=\frac{-B_{L}\sqrt{B_{L}^{2}+1}+arsinh\left(B_{L}\right)}{4A_{L}}-constant,$$

where *A_L_, B_L_, C_L_* and *P2_y_* are unknowns. The system of equations was solved by Newton’s method, as available in the open source “eigen library” of C++ for linear algebra. Similar equations hold for the right-hand side of the curvature.

### 2.5. Applicator Position and Orientation

So far, P1–P3 determined the curvature of the applicator. For a robust determination of the orientation and position of the applicator, including rotations around the coordinate axes, three additional points were used, such that the outer edges and the rotations of the applicator are correctly defined (Figure 4). Points P4 and P5 define the other two outer points of the dummy applicator. The software validates whether antenna sizes resulting from these coordinates match the real antenna dimensions. Point P6 is the central point on the dummy above P2 and these two points determine the rotation around the patient’s body axis (z-axis). For the orientation, rotation angles around the x- and y-axis with respect to P1 can be easily determined (see Figure 4).

### 2.6. Integration in Treatment Planning

**Figure 5:**
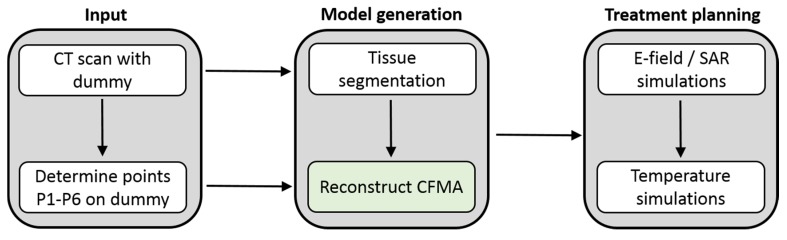
Integration of curved CFMA models into the treatment planning system. The modelling sequence for reconstruction of the CFMA is as follows: water bolus, rubber frame, electrodes, substrate, short circuit, exciting slot and coaxial cable.

The integration of curved CFMA models into the treatment planning system Plan2Heat is schematically represented in Figure 5. As input for the treatment planning a patient CT with a dummy applicator was required and the coordinates P1–P6 on the dummy were determined by the user. In the next step “Model generation” a patient model was created from the CT scan by segmenting it into fat, muscle, bone, lung and air using the CT Hounsfield Units. Additionally, the tumor can be delineated manually by a radiation oncologist if required for detailed evaluation of tumor heating, but this additional tumor delineation was not necessary for the purpose of the present study. This patient model was combined with the applicator model, which was modeled using a dedicated C++ module (represented by the green box in Figure 5) by assigning the required dielectric properties to the voxels corresponding to the different materials of the applicator, based on the placement information determined by the user-defined points P1–P6. The different layers of the bent CFMA (metal electrodes, substrate, rubber, water bolus) all follow the same curvature. The bolus membrane was not modelled separately, as justified by previous studies [9,21]. User-defined properties, such as water bolus thickness, applicator type and coordinates P1–P6, were imported via a parameter file.

For correct modelling of the applicator, the sequence of rasterizing the different structures is important. To ensure good contact between the water bolus and the patient, the water bolus was drawn thicker than required, prior to insertion of the patient model. Next, the curve of the rubber frame was modeled, followed by the two active electrodes and the shield electrode. The two active electrodes were first modeled as one single curve. Then the fluoroplastic substrate was modeled, which may in turn introduce small holes in the electrodes. This is corrected by an algorithm inserting missing electrode voxels. After this, the short-circuit (Figure 2) and the exciting slot were added. Finally, the coaxial feeding cable was modeled, consisting of an outer conductor, inner conductor and dielectric. This completes the model for the final “Treatment planning”, in which electromagnetic field and temperature simulations were performed.

### 2.7. Validation of the Curvature Approximation

A realistic treatment series of four sessions was mimicked using a standard Alderson radiation therapy phantom for which the 5H applicator was bent four times around the phantom’s chest by an experienced hyperthermia treatment operator, mimicking four separate consecutive “sessions”. After each “session” a photograph of the applicator was taken, from the same position relative to the applicator and each photograph was overlaid with a standard grid. The three points P1, P2 and P3 (Figure 4) were defined on the photograph and a line following the applicator treatment curvature was drawn. Additionally, 12 points were drawn that represent the treatment curvature, i.e., the part of the curve between two successive points can be represented by a straight line segment. Treatment curvatures were aligned such that the variance was minimized and the overall maximum difference and the average maximum difference between the first and the remaining sessions were determined. A CT scan with the dummy applicator was made to reconstruct a treatment planning model and the modelled curvature was compared to the treatment curvature.

Additionally, to validate the approximation of the applicator curvature by two polynomial functions, the reconstructed curvature was determined using points P1–P3 and compared to the real curvature for each of the four mimicked treatment session. The maximum and average distance between the real and reconstructed curves was determined by subtraction of the 9 points (i.e., 12 points excluding fixed points P1–P3).

Next, the approximation of the applicator curvature was validated after clinical treatment sessions. This was done once for each of the five applicator sizes 1H–5H. Again, photographs were taken and the treatment curvature was drawn on the photograph and reconstructed. A number of 6–12 points were drawn that represent the curvature and the maximum and average distance between the real and reconstructed curves was determined, as described above.

### 2.8. Superficial Hyperthermia Treatment Planning

To show the possible clinical application of the developed method, treatment planning was performed for a patient with recurrent breast cancer. An anonymized planning CT scan (resolution 1 × 1 × 1.25 mm^3^) of a patient with a dummy applicator was used to reconstruct a segmented patient model. This model was combined with the CFMA model reconstructed using the method described above, at a resolution of at 2 × 1 × 2 mm^3^. The electromagnetic field distribution in the patient was calculated using the finite difference time domain method [28]. Perfectly matched layer (PML) boundary conditions were used to avoid reflections at the model boundaries [29]. From the electric field distribution, the SAR was calculated using

(20) $$\mathrm{SAR}=\;\frac{\sigma }{2\rho }{\left\|\vec{E}\right\|}^{2},$$

with *σ* (S m^−1^) the electrical conductivity and *ρ* (kg m^−3^) the tissue density. The temperature distribution was calculated using Pennes’ bioheat equation [30]:

(21) $$c\rho \frac{\partial T}{\partial t}=\nabla \cdot \left(k_{tis}\nabla T\right)-c_{b}W_{b}\left(T-T_{art}\right)+\rho \cdot SAR,$$

with *c* the specific heat capacity (J kg^−1^ °C^−1^). The heat conduction in tissue is represented by the term $\nabla \cdot \left(k_{tis}\nabla T\right)$, with *k_tis_* (W m^−1^ °C^−1^) the thermal conductivity. The second term models the perfusion, with *c_b_* the specific heat capacity of blood, *W_b_* (kg m^−3^ s^−1^) the volumetric perfusion rate and *T_art_* the local arterial or body core temperature. Tissue properties were taken from the literature [26,27]. The water bolus was modelled as a fixed temperature boundary condition (Dirichlet). Steady-state tissue temperature distributions for two different water bolus temperatures (43 °C and 40 °C) were calculated. Absorbed power was scaled such that the overall maximum tissue temperature did not exceed 44 °C, which is the maximum tissue temperature allowed in the clinic during superficial hyperthermia to avoid thermal damage [5]. A target area of 15 cm × 15 cm was assumed and different target depths of 1, 2 and 3 cm from the skin were considered. Temperature volume histograms were determined for each target depth and the T90 values were compared. The T90 is the temperature at least achieved in 90% of the target volume, and is an important predictive parameter for hyperthermia treatment outcome.

## 3. Results

### 3.1. Curvature Reconstruction

**Figure 6:**
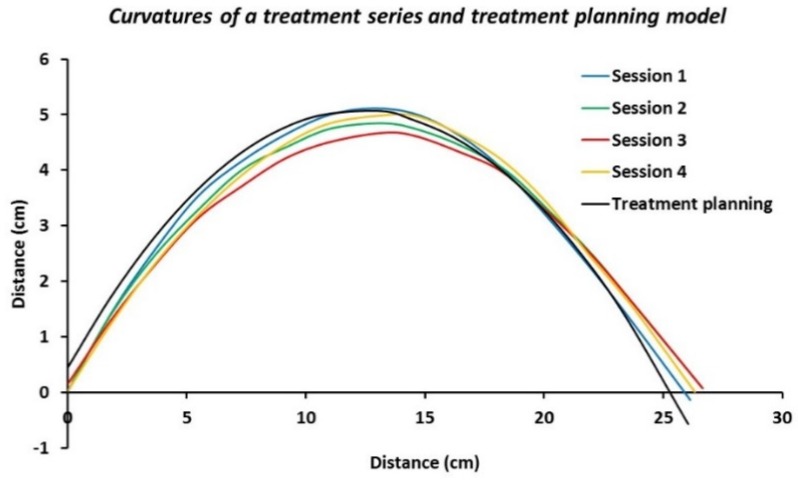
Curvatures of the 5H applicator positioned on the chest of the Alderson radiation therapy phantom for four consecutive mimicked treatment sessions, as well as the modeled curvature from the reconstructed treatment planning model.

Figure 6 shows the real curvatures of a mimicked treatment series of four sessions using the Alderson phantom for which the 5H applicator was bent four times around the phantom’s chest, as well as the modeled curvature from the reconstructed treatment planning model. The overall maximum variation and the average maximum variation in curvature between Session 1 (i.e., the reference session) and Sessions 2–4 were found to be 4.60 mm and 3.67 ± 0.69 mm, respectively. The variation ranged between 2.98 and 4.60 mm. The maximum deviation between the modeled curvature and Session 1 was 4.35 mm, which falls well within the range of the inter-session variation. A slight translation could be necessary to also closely match the curvature position to the model curvature, e.g., for Session 3. This supports the need to ensure reproducible positioning for successive treatment sessions; see Discussion Section.

Maximum and average deviations between the real applicator curvature and the curvature estimation by two polynomial functions for each session are shown in Table 1. The average deviation along the curvature was typically less than 1.5 mm and the maximum deviation was at most 4 mm, varying between 2.47 mm and 4.12 mm.

Maximum and average deviations between the real applicator curvature after a clinical treatment session and the estimated curvature by two polynomial functions for each of the five applicator sizes 1H–5H, are shown in Table 2. Here, the average deviation along the curvature was typically less than 1 mm and the maximum deviation was less than 2 mm, varying between 0.48 mm and 1.98 mm.

Comparing the deviations between the real and estimated applicator curvatures to the variation between treatment sessions, we can conclude that the error of approximating the applicator curvature by two polynomial functions is typically smaller than the variation in treatment curvature during a treatment series and thus that this reconstruction method is suitable for clinical use.

### 3.2. Treatment Planning Example

**Figure 7:**
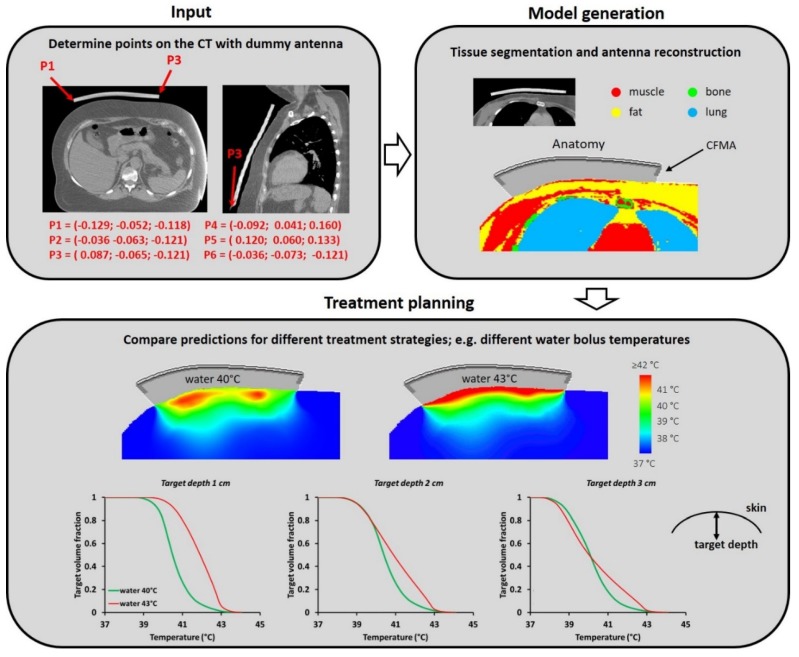
Illustration of the process of treatment planning for superficial hyperthermia, using the method described in this paper. Location of reference points P1–P6 is given in Figure 4.

Figure 7 shows an illustration of the process of treatment planning for superficial hyperthermia, using the method described in this paper. The coordinates of points P1–P6 are selected manually on the CT scan with the dummy applicator, an action which takes only up to 5 min. After tissue segmentation and reconstruction of the 3H CFMA based on the selected points, a patient and applicator model for treatment planning were obtained within less than 5 min. Using this model the electric field distribution in the patient was calculated on the GPU, which took ~30 min for 50,000 time steps (computational volume 223 × 205 × 216 voxels, plus 16 layers PML) on a standard PC with an NVIDIA^®^ GeForce^®^ GTX 760 graphics card. This allows to compare predictions for different treatment strategies, e.g., different water bolus temperatures. Steady-state thermal simulations can be performed within 1 min on the GPU. As an example, temperature predictions with a water bolus temperature of 40 °C and 43 °C are shown. The amount of applied power was scaled such that the maximum tissue temperature does not exceed the maximum allowed tissue temperature of 44 °C. The total amount of power absorbed in the patient was 47.5 W and 37 W, for a bolus temperature of 40 °C and 43 °C, respectively. With a bolus temperature of 40 °C instead of 43 °C, the skin temperature is reduced, thereby shifting the maximum tissue temperature to a slightly deeper location (approximately 1 cm below the skin), and increasing the effective heating depth. The 40 °C water bolus therefore also allows a higher amount of applied power. Whether a relatively high or low bolus temperature is more effective depends on the target depth. The T90 is an important parameter for treatment quality and the temperature volume histograms show that for a target depth smaller than 2 cm, a 43 °C water bolus yields a higher T90 than a 40 °C water bolus. When the target depth is larger than 2 cm, 40 °C becomes more effective; predicted T90 values for a target depth of 3 cm are 38.55 °C and 38.81 °C, for a water temperature of 43 °C and 40 °C, respectively.

This type of 3D temperature predictions become possible with the method described in this paper. Accompanying the limited thermometry available during clinical treatments, these predictions can be very helpful to further optimize superficial hyperthermia treatments for individual patients.

## 4. Discussion

This paper presented a new method to reconstruct the clinical position, curvature and orientation of a class of bendable hyperthermia applicators (CFMAs). The method reconstructs the applicator based on only six user-defined coordinates selected manually on a dummy applicator on a standard CT scan, and the deviation along the curvature was typically less than 1.5 mm, which is less than the clinical variation in curvature among successive treatment sessions (~5 mm). The method is user-friendly and suitable for routine application in clinical patient-specific treatment planning, as was illustrated by a treatment planning example. For clinical treatment planning the tumor should also be delineated manually by a radiation oncologist, similar to treatment planning for locoregional hyperthermia and radiotherapy, which allows detailed evaluation and optimization of tumor heating. The tumor volume is then considered a separate tissue type to which adequate dielectric and thermal properties are assigned. The developed reconstruction method could also be applicable to other flexible or curved hyperthermia applicators with similar (mechanical) properties, such as the 70 MHz CFMA (SRPC “Istok”, Moscow). 

The small deviations in curvature we encountered will not have a significant impact on the SAR and temperature distributions, since from previous experiments we know that a strong variation in curvature is required to significantly influence the effective heating depth (EHD). The EHD is ~1.5 cm for a straight CFMA [9,19], and this increases with only 1–2 mm for a mild curvature (κ = 3.98 m^−1^) [19,21]. An approximately three times sharper curvature of 11.21 m^−1^ yields a substantial increase in EHD of ~1 cm [19,21]. Therefore, a small deviation in the curvature description of only a few mm is not expected to have a clinically relevant influence on the SAR pattern.

Routine clinical application of hyperthermia treatment planning is now applied for loco-regional hyperthermia treatment of deep-seated tumors treated with phased arrays of RF applicators, for which the position of the patient anatomy within the applicator array is reconstructed with the required spatial resolution of ±1 cm at ~70 MHz [31]. Routine application of treatment planning is lagging behind for hyperthermia of superficially located tumors due to the absence of sufficiently reliable reconstruction methods of the applicator position with respect to the anatomy, which require a five-fold better spatial resolution at the frequencies used for superficial hyperthermia (typically 434 and 915 MHz). This reconstruction method allows clinical use of hyperthermia treatment planning to further optimize treatment quality for superficial heating, which is important in view of the thermal dose–effect relationship. As illustrated, simulations can help to optimize treatment parameters, such as the water bolus temperature and applied power. The water bolus temperature is an important parameter for superficial tumor heating, since it determines the thermal penetration depth and thereby the heating effectiveness [32,33]. Since the skin is often also part of the target region, therapeutic skin temperatures should still be guaranteed, which indicates an optimal bolus temperature, depending also on the tumor depth. Simulations are an effective tool to optimize such treatment parameters for individual patients.

Furthermore, treatment planning can also be applied to determine whether effective heating of a specified tumor target with relatively deep infiltration is feasible with the available hyperthermia applicator; i.e., whether sufficient target coverage will be realized at depth. According to the hyperthermia guidelines published by the European Society of Hyperthermic Oncology, superficial heating can be used for lesions up to 4 cm depth, based on the penetration depth of electromagnetic energy at the applied frequencies [5]. However, heating up to a slightly larger depth might be feasible since bending a CFMA increases the penetration depth [21,34]. Simulations might help to determine whether superficial hyperthermia is adequate or whether a system with a lower operating frequency, and thus a larger penetration depth, should be applied, e.g., a 70 MHz CFMA [35] or the 70 MHz AMC-2 system [36]. These systems have been developed for heating superficial tumors with deep infiltration.

Adequate applicator positioning is important, as also indicated in a volunteer study by Arunachalam et al., which demonstrated minimal displacement over treatment for a curved thermobrachytherapy surface applicator [37]. For reliable treatment planning with the type of applicators evaluated in the present study, the treatment position and orientation of the applicator during the successive treatment sessions of a patient should be similar to the position and orientation of the dummy applicator on the pre-treatment CT scan. Reproducible positioning of the applicator at the start of each treatment session is thus essential. In our current clinical practice, where treatment planning was not yet available because of the lack of an applicator reconstruction method for curved applicators, applicators are positioned manually by eye, such that the tumor area is covered by the EFS region of the applicator. Reproducibility can be improved by using three or four temporary tattoos or other markers indicating the corners of the applicator. If necessary, additional laser-aided positioning can be applied to improve the positioning accuracy, as commonly used in radiotherapy. More sophisticated techniques capable of on-line position tracking also could be explored to ensure reproducible positioning, as for example used in clinical radiotherapy to improve the set-up accuracy by optically monitoring the patient’s surface and comparing it to the planned reference surface [38]. Such a position tracking system would allow both reproducible positioning as well as on-line capturing of the outer shape of the antenna and the exact antenna position for treatment planning when the coordinates of the six points required for antenna reconstruction are transmitted to the planning software. An additional advantage of using a position tracking system would be the possibility of real-time feedback on the positioning error, which would allow more accurate repositioning in successive treatment sessions. Evaluation of different methods for accurate and reproducible positioning is a subject of ongoing research.

## 5. Conclusions

The developed method is the first to allow reconstruction of CFMAs for hyperthermia treatment planning. The uncertainty in the approximation of the applicator curvature is typically less than 4 mm. As part of the clinically used treatment planning software Plan2Heat, the integrated module can be applied for routine superficial hyperthermia treatment planning to further improve treatment quality, for example by optimizing the water bolus temperature for patient-specific tumor depths. Predictive parameters for hyperthermia treatment outcome can easily be evaluated and compared for various input parameters.

## Figures and Tables

**Table 1:** Average and maximum deviations between the real and estimated applicator curvature for a mimicked treatment series of four sessions for the 5H applicator positioned on the chest of the Alderson radiation therapy phantom.

Session	Avg. Deviation (mm)	Max. Deviation (mm)
**1**	1.47 ± 1.45	4.12
**2**	1.53 ± 1.01	3.25
**3**	0.81 ± 0.85	2.47
**4**	1.10 ± 0.99	3.43

**Table 2:** Average and maximum deviations between the real applicator curvature after a treatment session and the estimated curvature.

Applicator	Avg. Deviation (mm)	Max. Deviation (mm)
**1H**	0.99 ± 0.43	1.5
**2H**	0.28 ± 0.19	0.48
**3H**	0.96 ± 0.68	1.98
**4H**	0.59 ± 0.37	1.21
**5H**	0.68 ± 0.46	1.72

## References

[1] Wust P., Hildebrandt B., Sreenivasa G., Rau B., Gellermann J., Riess H., Felix R., Schlag P.M. (2002). Hyperthermia in combined treatment of cancer. Lancet Oncol..

[2] Vernon C.C., Hand J.W., Field S.B., Machin D., Whaley J.B., Van der Zee J., van Putten W.L.J., Van Rhoon G.C., van Dijk J.D.P., González D. (1996). Radiotherapy with or without hyperthermia in the treatment of superficial localized breast cancer: Results from five randomized controlled trials. International Collaborative Hyperthermia Group. Int. J. Radiat. Oncol. Biol. Phys..

[3] Bakker A., Holman R., Rodrigues D.B., Dobsicek Trefna H., Stauffer P.R., van Tienhoven G., Rasch C.R.N., Crezee H. (2018). Analysis of clinical data to determine the minimum number of sensors required for adequate skin temperature monitoring of superficial hyperthermia treatments. Int. J. Hyperth..

[4] Bakker A., Van der Zee J., van tienhoven G., Kok H.P., Rasch C.R.N., Crezee H. (2019). Temperature and thermal dose during radiotherapy and hyperthermia for recurrent breast cancer are related to clinical outcome and thermal toxicity: A systematic review. Int. J. Hyperth..

[5] Trefna H.D., Crezee H., Schmidt M., Marder D., Lamprecht U., Ehmann M., Hartmann J., Nadobny J., Gellermann J., van Holthe N. (2017). Quality assurance guidelines for superficial hyperthermia clinical trials: I. Clinical requirements. Int. J. Hyperth..

[6] Kok H.P., Wust P., Stauffer P.R., Bardati F., van Rhoon G.C., Crezee J. (2015). Current state of the art of regional hyperthermia treatment planning: A review. Radiat. Oncol..

[7] Prasad B., Kim J.K., Kim S. (2019). Role of Simulations in the Treatment Planning of Radiofrequency Hyperthermia Therapy in Clinics. J. Oncol..

[8] Kok H.P., Crezee J. (2017). A comparison of the heating characteristics of capacitive and radiative superficial hyperthermia. Int. J. Hyperth..

[9] Kok H.P., De Greef M., Correia D., Zum Vörde Sive Vörding P.J., Van Stam G., Gelvich E.A., Bel A., Crezee J. (2009). FDTD simulations to assess the performance of CFMA-434 applicators for superficial hyperthermia. Int. J. Hyperth..

[10] De Bruijne M., Wielheesen D.H., Van der Zee J., Chavannes N., Van Rhoon G.C. (2007). Benefits of superficial hyperthermia treatment planning: Five case studies. Int. J. Hyperth..

[11] Trujillo-Romero C.J., Paulides M.M., Drizdal T., van Rhoon G.C. (2015). Impact of silicone and metal port-a-cath implants on superficial hyperthermia treatment quality. Int. J. Hyperth..

[12] Bakker A., Kolff M.W., Holman R., van Leeuwen C.M., Korshuize-van Straten L., de Kroon-Oldenhof R., Rasch C.R.N., van Tienhoven G., Crezee H. (2017). Thermal Skin Damage during Reirradiation and Hyperthermia Is Time-Temperature Dependent. Int. J. Radiat. Oncol. Biol. Phys..

[13] Rietveld P.J., van Putten W.L., Van der Zee J., Van Rhoon G.C. (1999). Comparison of the clinical effectiveness of the 433 MHz Lucite cone applicator with that of a conventional waveguide applicator in applications of superficial hyperthermia. Int. J. Radiat. Oncol. Biol. Phys..

[14] Van Rhoon G.C., Rietveld P.J., van der Zee J. (1998). A 433 MHz Lucite cone waveguide applicator for superficial hyperthermia. Int. J. Hyperth..

[15] Puric E., Heuberger J., Lomax N., Timm O., Bodis S. (2009). The Benefit of Thermoradiotherapy in the Treatment of Superficially Localized Tumors: Experience with Bsd 500 Microwave Hyperthermia System. Strahlenther. Onkol..

[16] Johnson J.E., Neuman D.G., Maccarini P.F., Juang T., Stauffer P.R., Turner P. (2006). Evaluation of a dual-arm Archimedean spiral array for microwave hyperthermia. Int. J. Hyperth..

[17] Gelvich E.A., Mazokhin V.N. (2002). Contact flexible microstrip applicators (CFMA) in a range from microwaves up to short waves. IEEE Trans. Biomed. Eng..

[18] Gabriele P., Ferrara T., Baiotto B., Garibaldi E., Marini P.G., Penduzzu G., Giovannini V., Bardati F., Guiot C. (2009). Radio hyperthermia for re-treatment of superficial tumours. Int. J. Hyperth..

[19] Lamaitre G., Van Dijk J.D.P., Gelvich E.A., Wiersma J., Schneider C.J. (1996). SAR characteristics of three types of Contact Flexible Microstrip Applicators for superficial hyperthermia. Int. J. Hyperth..

[20] Florack L.M.J., Ter Haar Romeney B.M., Koenderink J.J., Viergever M.A. (1993). Cartesian Differential Invariants in Scale-Space. J. Math. Imaging Vis..

[21] Kok H.P., Correia D., De Greef M., Van Stam G., Bel A., Crezee J. (2010). SAR deposition by curved CFMA-434 applicators for superficial hyperthermia: Measurements and simulations. Int. J. Hyperth..

[22] Linthorst M., Drizdal T., Joosten H., van Rhoon G.C., van der Zee J. (2011). Procedure for creating a three-dimensional (3D) model for superficial hyperthermia treatment planning. Strahlenther. Onkol..

[23] Drizdal T., Paulides M.M., Linthorst M., van Rhoon G.C. (2012). Reconstruction of applicator positions from multiple-view images for accurate superficial hyperthermia treatment planning. Phys. Med. Biol..

[24] Kok H.P., Kotte A.N.T.J., Crezee J. (2017). Planning, optimisation and evaluation of hyperthermia treatments. Int. J. Hyperth..

[25] Hornsleth S.N., Mella O., Dahl O., Franconi C., Arcangeli G., Cavaliere R. (1996). A new segmentation algorithm for finite difference based treatment planning systems. Hyperthermic Oncology.

[26] Gabriel C., Gabriel S., Corthout E. (1996). The dielectric properties of biological tissues: I. Literature survey. Phys. Med. Biol..

[27] ESHO Taskgroup Committee (1992). Treatment Planning and Modelling in Hyperthermia, a Task Group Report of the European Society for Hyperthermic Oncology.

[28] Taflove A., Hagness S.C. (2000). Computational Electrodynamics.

[29] Berenger J.P. (1994). A Perfectly Matched Layer for the Absorption of Electromagnetic-Waves. J. Comput. Phys..

[30] Pennes H.H. (1948). Analysis of tissue and arterial blood temperatures in the resting human forearm. J. Appl. Physiol..

[31] Canters R.A., Franckena M., Paulides M.M., Van Rhoon G.C. (2009). Patient positioning in deep hyperthermia: Influences of inaccuracies, signal correction possibilities and optimization potential. Phys. Med. Biol..

[32] Van der Gaag M.L., de Bruijne M., Samaras T., Van der Zee J., Van Rhoon G.C. (2006). Development of a guideline for the water bolus temperature in superficial hyperthermia. Int. J. Hyperth..

[33] Arunachalam K., Maccarini P.F., Craciunescu O.I., Schlorff J.L., Stauffer P.R. (2010). Thermal characteristics of thermobrachytherapy surface applicators for treating chest wall recurrence. Phys. Med. Biol..

[34] Correia D., Kok H.P., De Greef M., Bel A., Van Wieringen N., Crezee J. (2009). Body conformal antennas for superficial hyperthermia: The impact of bending Contact Flexible Microstrip Applicators on their electromagnetic behavior. IEEE Trans. Biomed. Eng..

[35] Van Wieringen N., Wiersma J., Zum Vörde Sive Vörding P.J., Oldenborg S., Gelvich E.A., Mazokhin V.N., Van Dijk J.D.P., Crezee H. (2009). Characteristics and performance evaluation of the capacitive Contact Flexible Microstrip Applicator operating at 70 MHz for external hyperthermia. Int. J. Hyperth..

[36] Van Stam G., Kok H.P., Hulshof M., Kolff M.W., van Tienhoven G., Sijbrands J., Bakker A., Zum Vorde Sive Vording P.J., Oldenborg S., de Greef M. (2017). A flexible 70 MHz phase-controlled double waveguide system for hyperthermia treatment of superficial tumours with deep infiltration. Int. J. Hyperth..

[37] Arunachalam K., Craciunescu O.I., Markewitz E.J., Maccarini P.F., Schlorff J.L., Stauffer P.R. (2012). Preclinical assessment of comfort and secure fit of thermobrachytherapy surface applicator (TBSA) on volunteer subjects. J. Appl. Clin. Med. Phys..

[38] Laaksomaa M., Sarudis S., Rossi M., Lehtonen T., Pehkonen J., Remes J., Luukkanen H., Skytta T., Kapanen M. (2019). AlignRT^®^ and Catalyst^TM^ in whole-breast radiotherapy with DIBH: Is IGRT still needed?. J. Appl. Clin. Med. Phys..

